# Anticonvulsant effects of iridoid glycosides fraction purified from *Feretia apodanthera* Del. (Rubiaceae) in experimental mice models of generalized tonic-clonic seizures

**DOI:** 10.1186/s12906-016-1269-8

**Published:** 2016-08-12

**Authors:** Germain Sotoing Taiwe, Bernard Dabole, Thierry Bang Tchoya, Joseph Renaud Menanga, Paul Désiré Djomeni Dzeufiet, Michel De Waard

**Affiliations:** 1 Department of Zoology and Animal Physiology, Faculty of Science, University of Buea, Buea, Cameroon; 2 Department of Chemistry, Faculty of Science, University of Maroua, Maroua, Cameroon; 3 Department of Animal Biology and Physiology, Faculty of Science, University of Yaoundé I, Yaoundé, Cameroon; 4 Institut du Thorax, Inserm UMR 1087/CNRS UMR 6291, Nante, France; 5 University of Nante, Nante, France; 6 Smartox Biotechnology, Saint Martin d’Hères, France

**Keywords:** Iridoid glycosides, Tonic-clonic seizures, Kindling development, Oxidative stress, Antiepileptic drugs

## Abstract

**Background:**

Despite the increasing number and variety of antiepileptic drugs, nearly 30 % of epileptic patients who receive appropriate medical attention have persisting seizures. Anticonvulsant activity has been demonstrated for different iridoid glycoside-rich plant extracts. This study was designed to investigate the anticonvulsant effects of iridoid glycosides purified from *Feretia apodanthera* and to explore the possible mechanisms involved in antiepileptic activity.

**Methods:**

The anticonvulsant effects of iridoid glycosides extracts were investigated against 2.7 mg/kg bicuculline- and 70 mg/kg pentylenetetrazole-induced convulsions. The behavioural and electroencephalographic manifestations of 50 mg/kg pentylenetetrazole-induced seizures in mice as a model of generalized tonic-clonic seizures were also evaluated. Finally, the extracts were tested on the course of kindling development, kindled-seizures and oxidative stress markers in 30 mg/kg pentylenetetrazole-kindled mice. Their effects on brain GABA content were also determined.

**Results:**

The iridoid glycosides (30–90 mg/kg) protected mice against bicuculline-induced motor seizures in all pre-treated animals. Behavioural seizures- and mortality-induced by 70 mg/kg pentylenetetrazole were strongly antagonized by the extracts (60–90 mg/kg). The number of crisis (n/20 min), the cumulative duration of crisis (sec/20 min), and the mean duration of crisis (sec) recorded in 50 mg/kg pentylenetetrazole-treated mice were significantly decreased in all pre-treated mice with the extracts (60–90 mg/kg). Administration of the extracts (30–90 mg/kg) significantly increased the latency to myoclonic jerks, clonic seizures as well as generalized tonic-clonic seizures, improved the seizure mean stage and decreased the number of myoclonic jerks in 30 mg/kg pentylenetetrazole-kindled mice. Pentylenetetrazole kindling induced significant oxidative stress and brain GABA content alteration that was reversed by pretreatment with the extracts in a dose-dependent manner.

**Conclusions:**

The results indicate that pretreatment with the iridoid glycosides extracts of *Feretia apodenthera* improves generalized tonic-clonic seizures-induced by chemo-convulsants, protects mice against kindling development and oxidative stress, and improves brain GABA content in pentylenetetrazole-kindled mice.

## Background

Epilepsy is a prevalent neurological disorder that seriously affects the living quality of more than 65 million people worldwide [1, 2]. It is well acknowledged that epileptic seizures are the result of instant abnormal hyper-synchronous electrical activity of neuronal networks originating locally from discharges of brain regions. Imbalance between excitation and inhibition appears as the main cause of these abnormal electrical activities [3–5]. Individual generalized seizure types include absence, myoclonic, tonic-clonic, atonic, tonic, and clonic symptoms. In many studies, it is not clear that “generalized seizure” is synonymous with generalized onset seizure, leading to some ambiguity in classification by seizure type [6]. Despite the increasing number and variety of antiepileptic drugs, nearly 30 % of epileptic patients who have been taken in charge medically have persistent seizures [7]. The pharmacological resistance of epilepsy remains therefore too widespread. Also, current treatments of epilepsy are not satisfactory in terms of drug-associated deleterious effects [8]. Further, adverse effects associated with antiepileptic drugs and recurrent seizures limit their use. The development of new, affordable and accessible pharmacological agents that can overcome these limitations has become a major goal in epilepsy research [9]. The plant kingdom has become a target of great interest and value in the search of new drugs and lead compounds to treat several neurological disorders, including epilepsy for many years [7, 10].

*Feretia apodanthera* Del. is a member of the Rubiaceae family. This plant is found mainly in the savanna regions of the West African coast. Preparations of the plant have been used in Cameroonian’s folk medicine to manage epilepsy, infantile convulsions, anxiety, psychoses, pain and inflammation for many years, and their efficacies are widely acclaimed among the rural communities of Northern Cameroon [11–15]. Previous studies in our laboratory found that intra-gastric administration of an aqueous extracts of *Feretia apodenthera* significantly reduced the progression of kindling-induced by pentylenetetrazol, and attenuated the oxidative stress and cognitive impairment in kindled-mice [10]. The aqueous extracts of *Feretia apodenthera* contained flavonoids, alkaloids, saponins, tannins, glycosides, anthraquinones and phenols, but not lipids [10]. Many of these compounds are used widely as food additives or in traditional medicine, prompting phytochemical investigations that have in turn uncovered a variety of flavonoids, alkaloids and iridoid glycosides [16–18]. Recent study showed that the treatment with cornel iridoid glycosides isolated from *Cornus officinalis* Sieb. et Zucc improved neurobehavioral deficits, decreased cerebral infarct size, reduced nitric oxide and inhibited nuclear factor kappa B expression in the brain of rats 24 h after focal cerebral ischemia [19, 20]. These results indicate that cornel iridoid glycosides may improve the microenvironment of the central nervous system by increasing growth/trophic factors and decreasing inflammation-related factors. Bailleul et al. [18] showed that the iridoid glycosides-rich extracts from *Feretia apodanthera* decreases the spontaneous motor activity and exploratory behaviour in mice, increases hexobarbital-mediated sleeping time in rats, attenuates the intensity of amphetamine-induced stereotypies in mice, and inhibited catalepsy induced by chlorpromazine rats. It also protected rat and rabbit, respectively, against maximal electroshock- and pentylenetetrazol-induced seizures [18].

In this manuscript, in order to understand the preliminary anticonvulsant mechanisms of action during epilepsy and/or epileptogenesis, we purified five iridoid glycosides from the stem barks of *Feretia apodanthera* using bio-guided fractionation, and we screened the effects of the mixture of these five iridoid glycosides on the central nervous system using experimental rodent models of epilepsy. The aim of this study was to assess the efficacy of iridoid glycoside extracts of *Feretia apodanthera* against convulsions induced by two chemo-convulsants, bicuculline and pentylenetetrazole, in mice as a model of generalized tonic-clonic seizures [21, 22]. We also evaluated the effects of this extract on the course of kindling development, kindling-induced oxidative stress markers and brain GABA content in pentylenetetrazol-kindled mice.

## Methods

### Plant material and purification of iridoid glycosides

**Fig. 1:**
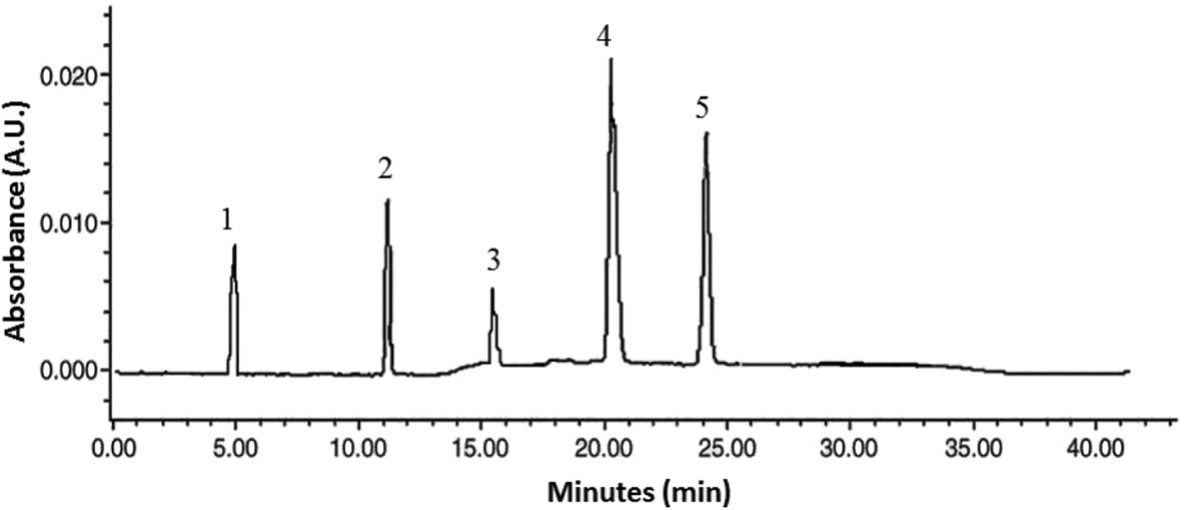
Typical RP-HPLC chromatograms at 240 nm for iridoid glycosides purified from the stem barks of *Feretia apodanthera*. (1) feretoside; (2): gardenoside; (3): geniposidic acid; (4): apodanthoside; (5): desacetylasperolosidic acid

**Fig. 2:**
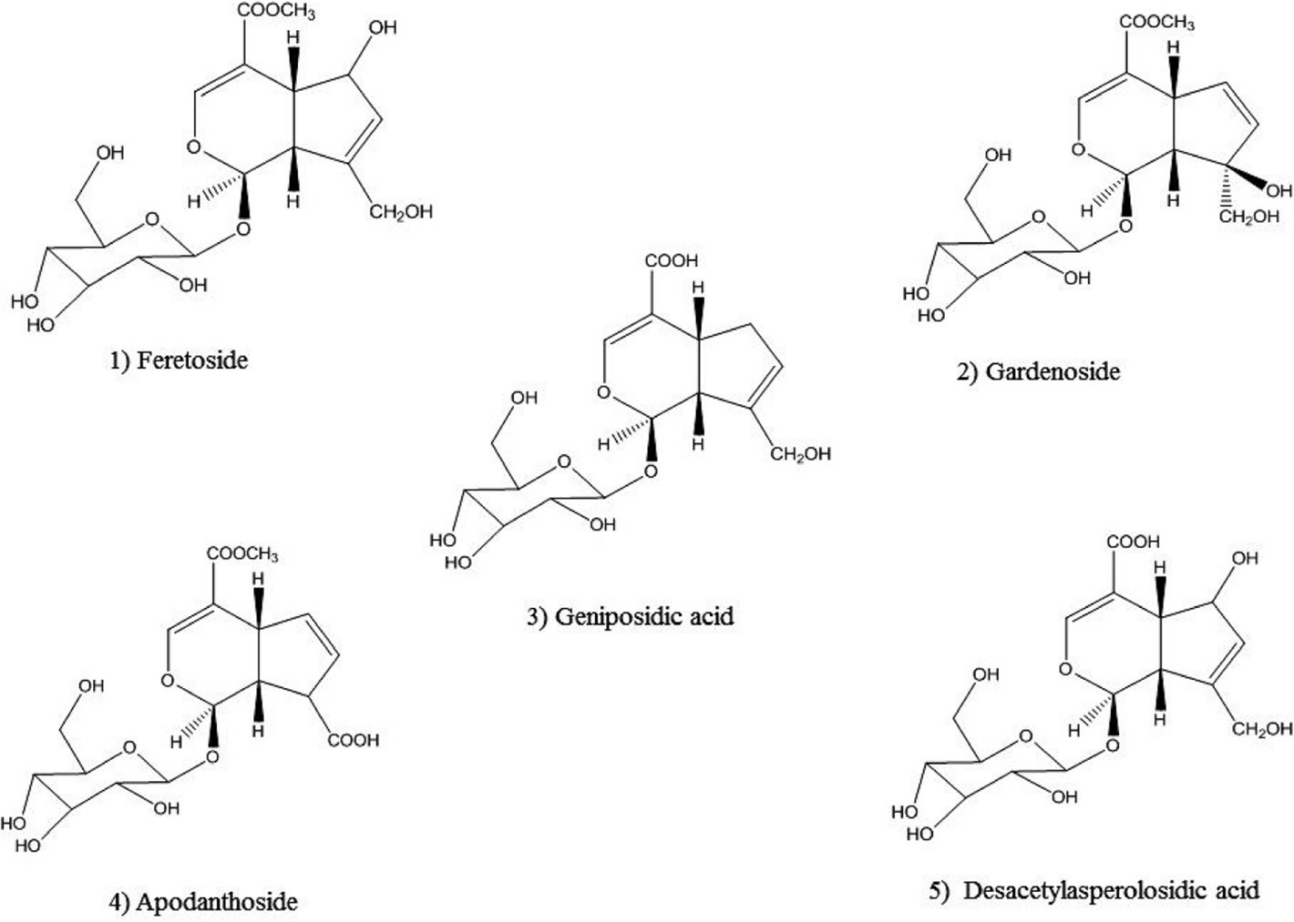
Chemical structures of five iridoid glycosides purified from the stem barks of Feretia apodanthera. (1) feretoside; (2): gardenoside; (3): geniposidic acid; (4): apodanthoside; (5): desacetylasperolosidic acid

The stem barks of *Feretia apodanthera* used in this study were harvested in the Mount Tenglin area of Pitoa (North Region of Cameroon, harvesting coordinates 9°25′17″ N and 13°27′2″ E). The plant collection was carried out on a private land, following the permission by the owner (Mr. Waziri Tshaolao, resident of Loumo quarter, Pitoa), to conduct the study on this site. The field studies did not involve endangered or protected species. The species was authenticated by the National Herbarium of Yaoundé (Cameroon), where a voucher was deposited (Sample Number 31225/HNC). Briefly, 500 g stem bark powder of *Feretia apodanthera* was decocted in 100 °C with 3 L water during 20 min and the solution was left to cool down. The procedure was repeated 3 times. The aqueous extract (2410 mL) was filtered, concentrated and subjected to XDA-16 macro-porous adsorptive resins chromatographic column (250 g, Ф 8 cm × 50 cm), which had been depurated with 95 % alcohol, with a flow rate of 2.0 mL/min. 600 mL distilled water was added to elute the iridoid glycoside extract. The end of the elution procedure was witnessed by a negative Molish reaction. Column was cleaned afterwards with 800 mL 50 % EtOH. Total iridoid glycoside extracts (27.9 g) was obtained after evaporation at 60 °C using a rotavapor. After, the extract was dissolved in distilled water (150 mL) and chromatographed on a silica gel column prewashed with 20 % EtOH (300 mL). The iridorid glycosides of *Feretia apodanthera* (IGEFA; 14.9 g) were obtained by using CHCl_3_-MeOH-H_2_O (43/37/20, vol/vol/vol) as eluant. The purities of iridoid glycosides were determined by RP-HPLC assay. The analyses were carried out on a HPLC system (Shimadzu-LC 20AT) equipped with UV detector, pressure controlled by prominence pump and operated by spinchrom Software. The HPLC separation method was developed on an Agilent reversed-phase octyldecyl silica column (TC-C18, 4.6 mm × 150 mm, 5 μm) and for elution of the constituents, a gradient of two solvents denoted A and B was employed. The mobile phase consisted of solvent A (water) and solvent B (acetonitrile) with the following gradient program: 30 % B in 0 ~ 5 min; 30 % ~ 35 % B in 5 ~ 15 min; 35 % ~ 60 % B in 15 ~ 20 min; 60 % B in 20 ~ 30 min. The flow rate was set at 1.0 mL/min, column temperature was kept at 30 °C and the injection volume was 20 μL. The sample was dissolved in a mixture of acetonitrile and water (30:70, v/v). Iridoid glycosides were detected at a wavelength of 240 nm (Fig. 1). The structures have been successfully elucidated by using homo- and heteronuclear two-dimensional nuclear magnetic resonance (NMR) techniques such as ^1^H-NMR, ^13^C-NMR, heteronuclear multiple quantum coherence (HMQC) and heteronuclear multiple bond correlation (HMBC) spectra, and mass spectrum [16–18]. The purity of IGEFA was 78.5 %, in which feretoside (1) accounted for 29 %, gardenoside (2) 18 %, geniposidic acid (3) 14 %, apodanthoside (4) 23 % and desacetylasperolosidic acid (5) 16 %, respectively (Fig. 2). The IGEFA was dissolved in saline 0.9 % containing dimethyl sulfoxyde 2 % (vehicle) at the appropriate concentrations as indicated in the various experiments and administered per os (p.o.) with a volume of 10 mL/kg.

### Chemicals

Bicuculline, clonazepam, diazepam, flumazenil, methyl-β-carboline-3-carboxylate (FG7142), pentylenetetrazole, sodium valproate, reduced glutathione, thiobarbituric acid, *n*-butanol, pyridine, sodium dodecyl sulphate, 5′5-dithiobis (2-nitrobenzoic acid) and trichloroacetic acid were obtained from Sigma, St. Louis, MO, USA. All other chemicals and reagents used in the brain γ-aminobutyric acid (GABA) content estimation were obtained from Sigma, St. Louis, MO, USA. Diazepam was obtained from Roche (France).

### Animals

Experiments were conducted on Swiss male mice (20–25 g) housed in appropriate cages with food and water *ad libitum*. They were kept in 12 h light/dark cycle. Each animal was used only once and was handled according to standard protocols for the use of laboratory animals. The investigation conforms to the Guide for the Care and Use of Laboratory Animal published by the US National Institutes of Health (NIH; publication No. 85-23, revised 1996) and received approval of the Cameroon National Ethical Committee (Yaounde, Cameroon) for animal handling and experimental procedure (Ref N°FW-IRB00001954). All efforts were made to minimize animal suffering and reduce the number of animals used.

### Pharmacological tests

#### Bicuculline-induced motor seizures

Mice were divided into six groups of eight mice and received orally different doses of IGEFA (15, 30, 60 and 90 mg/kg; per os (p.o.)), diazepam (positive control; 5 mg/kg, intraperitoneal (i.p.)) or vehicle (10 mL/kg; p.o.). One hour later, all animals were injected intraperitoneally with bicuculline (2.7 mg/kg; subcutaneously (s.c.)) and placed in isolated cages. Motor seizures, induced by bicuculline in mice, were visually observed by two independent investigators, unaware of the identity of the experimental groups. They were quantified in experimental and matched control mice using the following parameters: (a) the time to onset of the first seizure (either tonic or clonic); (b) the duration of the clonic and tonic components of seizures; (c) the number of motor seizures and (d) mortality. Clonic seizures consisted of rhythmic contractions of forelimbs and/or hindlimbs and/or the back muscles [23, 24]. A tonic seizure consisted of a rigid extension of the fore and/or hind limbs with or without loss of posture. The time of observation was 120 min [24, 25].

### Pentylenetetrazole-induced seizures

#### Experiment 1: acute administration of pentylenetetrazole at a dose of 70 mg/kg

Mice were divided in six groups of eight mice and received different treatments. Group I (negative control) was treated with vehicle (10 mL/kg p.o.). Groups II to V (test groups) were treated with 4 doses of IGEFA (15, 30, 60 and 90 mg/kg; p.o.). Group VI treated with clonazepam, 0.1 mg/kg i.p., was used as positive control. Tonic-clonic seizures were induced in mice by i.p. injection of 70 mg/kg pentylenetetrazole. The protective effect of the different treatments given 1 h before pentylenetetrazole injection was recorded. Animals that did not convulse within the 10 min of observation were qualified as protected [9].

### Involvement of the GABA_A_ receptor complex in pentylenetetrazole (70 mg/kg) test

We also studied the effects of a selective benzodiazepine receptor antagonist, flumazenil (1, 2, 3, 4, 5 and 6 mg/kg) and FG7142 (4, 6, 8, 10, 12 and 14 mg/kg), an inverse diazepam receptor agonist of the GABA_A_ receptor complex, on the anticonvulsant activity of IGEFA in order to investigate the probable involvement of benzodiazepine receptors [26]. In these antagonistic experiments, flumazenil and FG7142 were injected 15 min prior to the IGEFA (90 mg/kg) treatments.

### Experiment 2: acute administration of pentylenetetrazole at a dose of 50 mg/kg

#### Surgery

Animal surgery was done as previously described by Riban et al. [27]. Under general anesthesia (chloral hydrate 4 % in NaCl 0.9 %, 10 ml/kg i.p.) six groups of six mice were implanted stereotaxically with (i) two monopolar surface electrodes placed over the left and right frontoparietal cortex for pharmacological experiments or a bipolar electrode within the right frontal sensorimotor cortex (AP = +1.4 mm, ML = −1.6 mm, DV = −2 mm from Bregma) for signal analysis; (ii) a monopolar electrode placed over the cerebellum (reference electrode), and (iii) a bipolar electrode inserted into the hippocampus; with bregma as the reference [28]. The electrodes were made of stainless steel wire isolated by polyester (diameter, 0.125 mm). They were inserted in the skull above the cortex and the cerebellum. The bipolar electrode was formed of two twisted polyester insulated stainless steel wires. The implant assembly was affixed to the skull with dental acrylic. After the surgical implantation, mice were injected with antibiotics and housed individually in cages at least 14 days for recovery before the onset of experiments [27].

### Electroencephalographic recordings

Electroencephalograms (EEGs) were recorded in awake freely moving animals using a digital acquisition system (Biopac System, MP-100, Inc.). The signals were amplified and filtered (high pass filter 1 Hz/low pass filter 97 Hz), then digitized at a sampling rate of 256 Hz and recorded using AcqKnowledge® software version 3.2 (Biopac Students Lab PRO software). During the recording and stimulation sessions, the mice were continuously watched to detect changes in their posture and behaviour. All sessions did not exceed 3 h and were performed between 9:00 a.m. and 5:00 p.m [27]. Digital video recordings were made with a webcam (Orbit Logitech Quickcams) located inside the Faraday cage, and the animals’ behaviour was simultaneously recorded (for detailed information on surgery and procedures).

### Treatments and seizures induction

All pharmacological compounds were tested between the third and the sixth week following electrodes implantation. To test the effects of IGEFA on pentylenetetrazole-induced seizures, the animals were first divided into six groups of six mice each, and received different doses of IGEFA (15, 30, 60 and 90 mg/kg; p.o.), clonazepam (0.1 mg/kg, i.p.) or vehicle (10 mL/kg p.o.). They were then recorded for 60 min before the pentylenetetrazole injection (50 mg/kg, i.p.) and then for 120 min. The latency of first crisis, the cumulative duration of crisis, the mean duration of crisis and number of crisis were quantified.

### Histology

Upon completion of the experiments, all mice were injected with a lethal dose of pentobarbital (100 mg/kg, i.p.). Their brains were removed, frozen and cut in 20 μm sections using a cryostat. Histological analysis was performed following Cresyl Violet staining and each implantation site was localized with reference to the atlas of Paxinos and Watson [29].

### Experiment 3: chronic administration of pentylenetetrazole at a dose of 30 mg/kg

#### Experimental design

Animals were randomly divided into eight groups of six animals each. The first group received saline intraperitoneally while the second–seventh groups were administered pentylenetetrazole (30 mg/kg; i.p.) dissolved in saline on every second day (48 ± 2 h) [30]. One hour before administration of pentylenetetrazole, the first and second groups received vehicle (10 mL/kg), the third–sixth groups were administered IGEFA (15, 30, 60 and 90 mg/kg; p.o., respectively) orally through an intra-gastric feeding tube. Group seven animals were administered sodium valproate (300 mg/kg) intraperitoneally. Pentylenetetrazole and IGEFA were administered up to day 43 or until seizure stage 5 on two consecutive trials was achieved, whichever was earlier. Mice were observed for 30 min after the subconvulsant PTZ and seizure activity scored using a scoring system from 0 to 5. In group eighth and ninth, IGEFA (90 mg/kg) or sodium valproate, respectively, were administered alone to study any per se effects of the IGEFA or sodium valproate on behaviour and biochemical parameters. Group ten animals were administered vehicle and one hour later they received saline. Behavioural test were performed 24 h after the last administration of pentylenetetrazole. Following the behavioural evaluation, the animals were sacrificed and the whole brain was dissected for estimation of markers of oxidative stress.

### Kindling induction

For pentylenetetrazole kindling, a sub-convulsant dose of pentylenetetrazole (30 mg/kg, in a volume of 10 mL/kg) was injected intraperitoneally on every second day (i.e. day 1, day 3, day 5. . .). The first incidence of seizure with stage five was observed between day 35 (i.e. 18^th^ injection) and day 39 (i.e. 20^th^ injection). Pentylenetetrazole was administered up to day 43 (22^nd^ injection) or until seizure stage 5 on two consecutive trials was achieved, whichever was earlier. Seizure activity was evaluated for 30 min using the following scale [31]: Stage 0: no response; Stage 1: hyperactivity, vibrissae twitching; Stage 2: head nodding, head clonus and myoclonic jerk; Stage 3: unilateral forelimb clonus; Stage 4: rearing with bilateral forelimb clonus; Stage 5: generalized clonic-tonic seizures (or death within 30 min) with loss of writing reflex. The number of myoclonic jerks and the latencies to myoclonic jerks and generalized clonic-tonic seizures were recorded. Animals were considered kindled if they exhibited stage 5 of seizures on two consecutive trials. Animals were also observed for 24 h mortality.

### Tissue preparation

Following the behavioural testing, the mice were decapitated under ether anaesthesia and the brains were quickly removed, cleaned with ice-cold saline and stored at −80 °C.

The whole brain of each mouse was dissected out and divided into two cerebral hemispheres for biochemical estimations. From one half, 10 % (w/v) homogenate was prepared with ice-cold 0.1 M phosphate buffer (pH 7.4), and lipid peroxidation product, reduced glutathione and GABA concentration were assessed.

### Brain lipid peroxidation

Malondialdehyde, a measure of lipid peroxidation, was measured as described by Jainkang et al. [32]. The reagents acetic acid 1.5 mL (20 %) pH 3.5, 1.5 mL thiobarbituric acid (0.8 %) and 0.2 mL sodium dodecyl sulphate (8.1 %) were added to 0.1 mL of processed tissue samples, and then heated at 100 °C for 60 min. The mixture was cooled with tap water and 5 mL of *n*-butanol/pyridine (15:1), 1 mL of distilled water was added. The mixture was vortexed vigorously. After centrifugation at 4000 rpm for 10 min, the organic layer was separated and absorbance was measured at 532 nm using a spectrophotometer. The concentration of malondialdehyde is expressed as nmol/g tissue.

### Brain reduced glutathione

Brain reduced glutathione was measured according to the method of Ellman [33]. The homogenate was mixed with equal quantity of 10 % trichloroacetic acid (v/v) and centrifuged to separate the proteins. To 0.01 mL of this supernatant, 2 mL of phosphate buffer (pH 8.4), 0.5 mL of 5′5-dithiobis (2-nitrobenzoic acid) and 0.4 mL of double distilled water were added. The mixture was vortexed and the absorbance read at 412 nm within 15 min. The concentration of reduced glutathione was expressed as μg/g tissue.

### Brain GABA level

The brain GABA level was estimated in groups of mice. The measurement of GABA, based on the method of Lowe et al. [34], was carried out as follows. The brains were rapidly removed, blotted, weighed and taken in ice cold 5 mL trichloroacetic acid (10 % w/v), homogenized and centrifuged at 10,000 g for 10 min at 0 °C. A sample (0.1 mL) of tissue extract was taken in 0.2 mL of 0.14 M ninhydrin solution in 0.5 M carbonate-bicarbonate buffer (pH 9.9). This solution was kept in a water bath at 60 °C for 30 min then cooled and treated with 5 mL of copper tartrate reagent (0.16 % disodium carbonate and 0.03 % copper sulphate and 0.0329 % tartaric acid). After 10 min, the fluorescence reading was taken at 377/451 nm in a spectrofluorimeter. For GABA standards, different amounts (20, 40, 60, 80, 100 μg) mixed with 1.5 μM glutamic acid were dissolved in 0.1 mL 10 % trichloroacetic acid (w/v). GABA level was determined by the measurement of the formed fluorescent product resulting from the reaction of GABA with ninhydrin in an alkaline medium, in the presence of glutamate [35]. The GABA content in brain was expressed in μg/g of wet brain tissue.

### Acute toxicity test

The acute toxicity test for the IGEFA was carried out to evaluate any possible sign of toxicity. Mice of either sex were divided into control and test groups. The first group served as a normal control treated with vehicle. The IGEFA (5, 15, 30, 90, 180, 360, 720, 1440, 2880 and 5760 mg/kg) was administered orally to different groups of mice. After administration of these extracts, mice were allowed access to food and water *ad libitum* and behavioural parameters including convulsion, hyperactivity, sedation, grooming, loss of righting reflex, increased or decreased respiration, food and water intake and mortality were observed for a period of 14 days [36]. The dose of extract necessary to induce mortality by 50 % relative to the control value, called the median lethal dose (LD_50_) was estimated according to the method described by Litchfield and Wilcoxon [37].

### Data analysis

Data were expressed as mean ± standard error of the mean (S.E.M.) per group. The percentages of protection against chemical-induced seizures were measured and Fisher’s exact test (two-tailed) was used to compare percentages of protection. For the behavioural seizure in the bicuculline test, the control groups were compared to the extract-treated groups by the two-way repeated measures analysis of variance, followed by Newman-Keuls post hoc test. The effects of antiepileptic drugs were assessed by counting the number of hippocampal paroxysmal discharges in 20 min period post-injections. Data were expressed as mean ± standard error mean of percentage of cumulative duration, mean duration and number of crisis per 20 min periods, compared with vehicle condition. For each extract, the number of recorded seizures during the 20 min period post-injection was compared between doses using a two-way analysis of variance with repeated measures. Post-hoc comparisons versus vehicle conditions were performed using the Newman-Keuls test. The differences were considered significant at *p* < 0.05. In the acute toxicity test the median lethal dose (LD_50_) was estimated according to the method described by Litchfield and Wilcoxon [37].

## Results

### Effects of IGEFA on bicuculline-induced motor seizures in mice

In all mice, i.p. injection of bicuculline successfully induced motor seizures as assessed by the experimenter (Table 1). Several behavioural effects were observed after the administration of bicuculline at a dose of 2.7 mg/kg, including clonic and tonic components of seizures, ataxia and head weaving. The IGEFA antagonized bicuculline-induced motor seizures in all pretreated mice. Pretreatment with IGEFA in doses ranging from 15–90 mg/kg significantly influence the time to onset of the first seizure (either clonic [F(7, 52) = 114.62; *P* < 0.001] or tonic [F(7, 38) = 125.17; *P* < 0.001]), the duration of the clonic [F(7, 64) = 146.46; *P* < 0.001] and tonic [F(7, 29) = 132.74; *P* < 0.001] components of seizures, the number of motor seizures [F(7, 42) = 106.22; *P* < 0.001] and the incidence of death [F(7, 26) = 97.21; *P* < 0.001]. The durations of both tonic and clonic seizures were significantly reduced. A significant delay was found in the time to onset of clonic seizures. Diazepam (5 mg/kg), an anticonvulsant, protected the mice against bicuculline-induced motor seizures. Responses to bicuculline in mice treated with IGEFA were not different from those of diazepam-treated mice (Table 1).

**Table 1:** Effects of IGEFA or diazepam on bicuculline-induced motor seizures in mice

Treatments	Dose (mg/kg)	Behavioural seizures					Mortality
		Onset (min)		Duration (min)		Number of motor seizures	
		Tonus	Clonus	Tonus	Clonus		
Vehicle + Saline	– + –	–	–	–	–	–	–
Vehicle + Bic	– + 2.7	2.35 ± 0.21	6.15 ± 1.12	5.32 ± 1.47	69.32 ± 1.38	24.16 ± 1.14	8/8
IGEFA + Bic	15 + 2.7	4.12 ± 0.27	11.16 ± 1.13	4.21 ± 1.15	19.12 ± 1.54*	14.57 ± 1.26	1/8***
IGEFA + Bic	30 + 2.7	6.15 ± 1.26*	13.53 ± 1.04*	3.49 ± 1.26	16.35 ± 1.29*	12.32 ± 1.39*	1/8***
IGEFA + Bic	60 + 2.7	7.21 ± 1.23*	16.17 ± 1.18*	3.15 ± 1.13	12.49 ± 1.17*	11.49 ± 1.81*	0/8***
IGEFA + Bic	90 + 2.7	7.49 ± 1.49*	18.32 ± 1.36*	2.34 ± 1.52*	11.26 ± 1.27**	11.52 ± 1.16*	0/8***
Diazepam + Bic	5 + 2.7	8.27 ± 1.28**	21.42 ± 2.19**	1.26 ± 1.24*	11.32 ± 1.45**	10.26 ± 1.28*	0/8***

Results are expressed as mean ± S.E.M., for 8 animals, bicuculline (2.7 mg/kg; s.c.) was injected intraperitonealy in mice

*Bic* bicuculline, *IGEFA* iridoid glycosides extracts of *Feretia apodenthera*

*P < 0.05, **P < 0.01, ***P < 0.001 significantly different compared to the vehicle, data were analysed by two-way ANOVA, followed by Newman-Keuls post hoc test

### Pentylenetetrazole-induced seizures

#### Effects of IGEFA on acute administration of pentylenetetrazole at a dose of 70 mg/kg

**Fig. 3:**
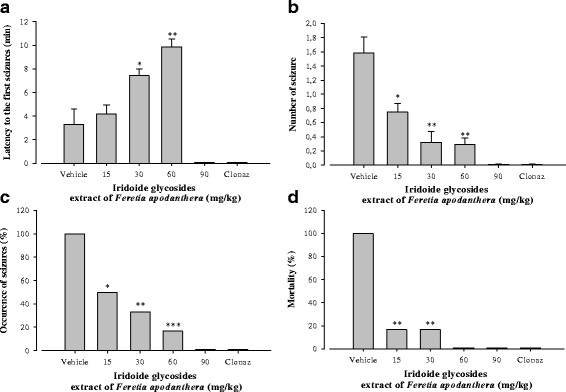
Effects of iridoids glycosids extract from the stem barks *Feretia apodanthera* on acute 70 mg/kg pentylenetetrazole-induced seizures in mice. Panel (**a**) latency to the first seizures, panel (**b**) number of seizure, panel (**c**) occurence of seizures (%), panel (**d**) mortality (%). *N* = 8 animals per dose. Data were analysis by Fisher Exact Test (two-tailed) for comparison of percentages or two-way repeated measures analysis of variance, followed by Newman-Keuls post hoc test for means comparison, ^*^ *P* < 0.05, ^**^ *P* < 0.01, ^***^ *P* < 0.001, significantly different compared to the vehicle; Clonaz, clonazepam 0.1 mg/kg. IGEFA, iridoids glycosids extract of *Feretia apodanthera*

Pentylenetetrazole (70 mg/kg) produced hind-limb tonic seizures in all the eight mice treated with vehicle. The IGEFA increased the latency to the first seizures from 3.3 ± 1.3 min to 7.4 ± 0.6 min [F(5, 28) = 92.5; *P* < 0.01] at the dose of 30 mg/kg, and from 3.3 ± 1.3 min to 9.9 ± 0.6 min [F(5, 28) = 92.5; *P* < 0.001] at the dose of 60 mg/kg (Fi. 3). The number of seizures was reduced in all the test groups as compared to negative control. The IGEFA dose-dependently protected animals against clonic seizures induced by pentylenetetrazole. At the dose of 15 mg/kg, the IGEFA protected 50 % (*P* < 0.05) of mice against seizures. The dose of 90 mg/kg provided a 100 % (*P* < 0.001) protection of mice. The percentage of mortality protection was highly significant for all tested groups when compared to negative control. Complete protection against mortality was achieved with 60 and 90 mg/kg IGEFA and clonazepam. These effects were comparable to that of clonazepam (0.1 mg/kg), a standard antiepileptic drug (Fig. 3).

### Involvement of the GABA_A_ receptor complex in pentylenetetrazole induced seizures

**Fig. 4:**
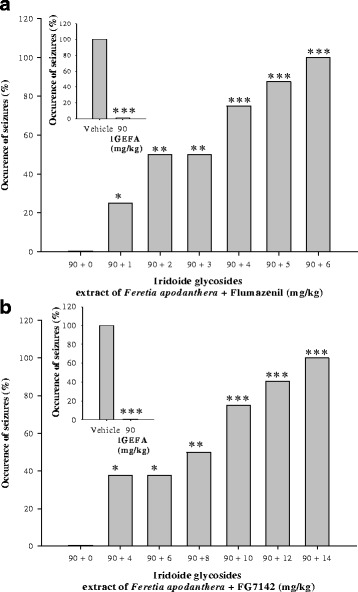
Effects of the coadministration of iridoids glycosids extract from the stem barks *Feretia apodanthera* (90 mg/kg) with flumazenil (1, 2, 3, 4, 5 and 6 mg/kg; Panel **a**) and FG7142 (4, 6, 8, 10, 12 and 14 mg/kg; Panel **b**), an inverse diazepam receptor agonist of the GABA_A_ receptor complex, on the convulsions induced in mice by acute 70 mg/kg pentylenetetrazole. *N* = 8 animals per dose. Data were analysis by Fisher Exact Test (two-tailed), ^*^ *P* < 0.05, ^**^ *P* < 0.01, ^***^ *P* < 0.001, significantly different compared to the vehicle; IGEFA, iridoids glycosids extract of *Feretia apodanthera*

The IGEFA, when administered alone at a dose of 90 mg/kg, protected all mice against pentylentetrazole-induced seizures. The anticonvulsant effects of the IGEFA were strongly antagonized by flumazenil (a specific diazepam receptor antagonist at the doses of 1, 2, 3, 4 5 and 6 mg/kg). This effect was statistically different from the data of vehicle [F(5, 34) = 72.4; *P* < 0.001] but did not exhibit differences with IGEFA administered at a dose of 15 mg/kg. In a similar manner, the anticonvulsant effect of the IGEFA (90 mg/kg) was strongly antagonized and abolished by FG7142 (a specific inverse diazepam receptor agonist at the doses of 4, 6, 8, 10, 12 and 14 mg/kg) (Fig. 4).

### Effects of IGEFA on acute administration of pentylenetetrazole at a dose of 50 mg/kg

**Fig. 5:**
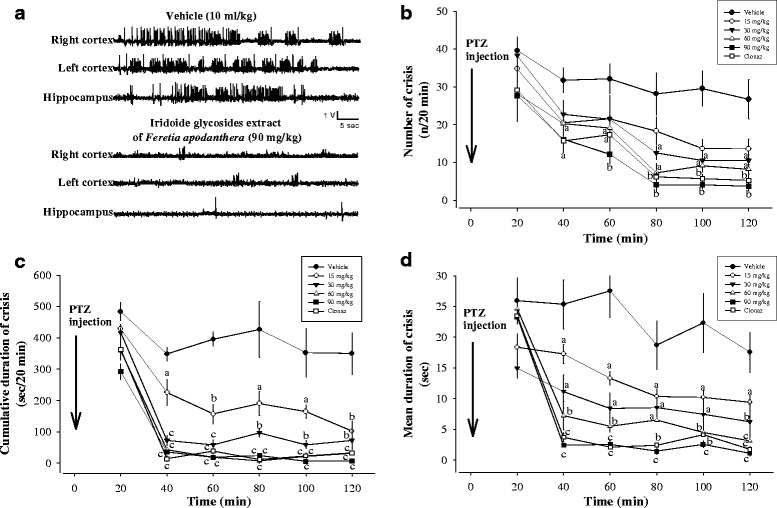
Effects of iridoids glycosids extract from the stem barks *Feretia apodanthera* on acute 50 mg/kg pentylenetetrazole-induced seizures in mice. Panel (**a**) hippocampal and cortical paroxysmal discharges, panel (**b**) number of crisis (n/20 min), panel (**c**) cumulative duration of crisis (sec/20 min), panel (**d**) mean duration of crisis (sec). *N* = 6 animals per dose. Data were analysis by two-way repeated measures analysis of variance, followed by Newman-Keuls post hoc test, ^a^ *P* < 0.05, ^b^ *P* < 0.01, ^c^ *P* < 0.001, significantly different compared to the vehicle; Clonaz, clonazepam 0.1 mg/kg. IGEFA, iridoids glycosids extract of *Feretia apodanthera*

**Fig. 6:**
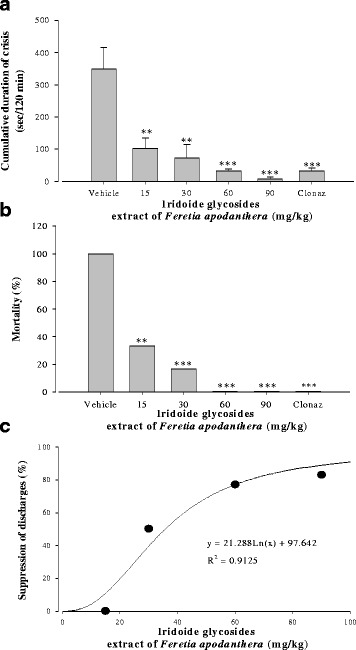
Effects of iridoids glycosids extract from the stem barks *Feretia apodanthera* on acute 50 mg/kg pentylenetetrazole-induced seizures in mice, continued. Panel (**a**) cumulative duration of crisis (sec/120 min), panel (**b**) mortality (%), panel (**c**) suppression of discharges (%). *N* = 6 animals per dose. Data were analysis by Fisher Exact Test (two-tailed) for comparison of percentages or two-way repeated measures analysis of variance, followed by Newman-Keuls post hoc test for means comparison; Clonaz, clonazepam 0.1 mg/kg. IGEFA, iridoids glycosids extract of *Feretia apodanthera*

Twenty min after pentylentetrazole (50 mg/kg) injection in animals, high voltage sharp waves (1500–4500 μV, 3–5 Hz) followed by higher frequency and low voltage rhythmic activity (10–14 Hz, 700–1100 μV) were recorded. These hippocampal and cortical paroxysmal discharges lasted between 20 and 60 s and their rate of occurrence was variable between vehicle-treated group or tested groups, with a maximum of one discharge every other minute (Fig. 5). Behavioural seizures with head nodding could be observed concomitantly with hippocampal and cortical paroxysmal discharges. However, some animals also displayed stereotyped behaviour, such as exploration or grooming. The antiepileptic effects of IGEFA were tested in pentylentetrazole-treated mice at the doses of 15, 30, 60 and 90 mg/kg. After pentylentetrazole administration at a dose of 50 mg/kg, vehicle-treated mice presented a high-voltage fast epileptiform activity with isolated spike-and-wave discharges (no significant difference by ANOVA). In vehicle-treated mice the number of crisis (n/20 min), the cumulative duration of crisis (sec/20 min), and the mean duration of crisis (sec) recorded were not significantly affected (Fig. 5). EEG activity was strongly impaired and animals showed signs of motor incapacitation. The acute administration of IGEFA at the dose of 90 significantly suppressed hippocampal and cortical paroxysmal discharges in a dose-dependent way (Figs. 5 and 6) and the number of crisis [F(5, 49) = 74.2; *p* < 0.001], the cumulative duration of crisis [F(5, 52) = 85.5; *p* < 0.001], and the mean duration of crisis [F(5, 34) = 92.5; *p* < 0.001] recorded were significantly decreased or partially blocked. No mortality of mice was recorded after administration of the IGEFA at the doses of 60 and 90 mg/kg (Figs. 5 and 6).

### Effects of IGEFA on chronic administration of pentylenetetrazole at a dose of 30 mg/kg

#### Effects on the development of pentylenetetrazole kindling

The data shown in Table 2 represent the effects of IGEFA or sodium valproate on treatment with a sub-convulsant dose (30 mg/kg) of pentylenetetrazole every 2 days. The data indicate that the development of kindled convulsions was directly proportional and cumulative with repeated exposure to pentylenetetrazole. Confirming previous studies (Taïwe et al., [36]), repeated administration of a sub-convulsant dose of pentylenetetrazole (30 mg/kg) on alternate days (for 30.00 ± 1.65 days, 15 injections) resulted in increasing convulsive activity leading to generalized clonic-tonic seizures (stage 5) [F(7, 28) = 124.8, *P* < 0.001] on two consecutive trials. Pretreatment with IGEFA at the doses of 15 mg/kg or vehicle did not modify the course of kindling induced by pentylenetetrazole. However, the higher doses of IGEFA (30, 60 and 90 mg/kg) suppressed the kindled seizure significantly (*P* < 0.001), as none of the animal could achieve stage 5 with 22 injections of pentylenetetrazole. The statistical analysis showed a significant difference in the development of kindling amongst the treated groups [F(7, 28) = 68.42, *p* < 0.001]. Sodium valproate (300 mg/kg) significantly (*p* < 0.05) delayed the course of development of kindling by pentylenetetrazole and provided complete protection against seizures (Table 2).

**Table 2:** Effects of IGEFA or sodium valproate on pentylentetrazol-induced kindling in mice

Treatments	Dose (mg/kg)		Behavioural seizure					
		kindling development (day)	Number	Duration (s)				Seizure mean stage
			Myoclonic jerks	Myoclonic jerks latency	Onset of clonic seizures	Latency to generalized clonic-tonic seizures	Duration of generalized clonic-tonic seizures	
Vehicle + Saline	– + –	–	–	–	–	–	–	–
Vehicle + PTZ	– + 30	30.00 ± 1.65	64.32 ± 2.45	45.45 ± 8.55	61.83 ± 8.61	185.45 ± 15.55	19.48 ± 1.32	4.00 ± 0.00
IGEFA + PTZ	15 + 30	32.21 ± 2.45	28.23 ± 2.14**	68.54 ± 5.25	75.16 ± 7.61	252.27 ± 24.14*	14.12 ± 1.25	2.33 ± 0.45*
IGEFA + PTZ	30 + 30	44.13 ± 2.28*	18.48 ± 2.13**	85.48 ± 4.47	185.21 ± 9.15***	324.15 ± 27.12***	8.24 ± 0.16**	2.16 ± 0.27*
IGEFA + PTZ	60 + 30	44.36 ± 2.32*	14.25 ± 2.34**	126.53 ± 8.15***	285.20 ± 7.62***	388.22 ± 35.26***	4.86 ± 0.24***	1.83 ± 0.28**
IGEFA + PTZ	90 + 30	43.72 ± 2.48*	8.45 ± 1.22***	173.62 ± 9.34***	371.52 ± 6.27***	495.48 ± 57.41***	3.29 ± 0.23***	1.33 ± 0.44**
SVA + PTZ	300 + 30	43.58 ± 2.34**	7.24 ± 1.56***	157.47 ± 8.52***	245.57 ± 6.45***	582.54 ± 48.36***	2.27 ± 0.15***	1.16 ± 0.27**
SVA + Saline	300 + –	–	–	–	–	–	–	–
IGEFA + Saline	90 + –	–	–	–	–	–	–	–

Results are expressed as mean ± S.E.M., for 6 animals. Data were analysis by two-way ANOVA, followed by Newman-Keuls post hoc test

*PTZ* pentylenetetrazol, *SVA* sodium valproate, *IGEFA* iridoid glycosides extracts of *Feretia apodenthera*

*P < 0.05, **P < 0.01, ***P < 0.001, significantly different compared to the vehicle-treated pentylentetrazole mice

### Effects of IGEFA on seizures in pentylenetetrazole kindled mice

As shown in Table 2, IGFA administered orally at a dose of 60–90 mg/kg suppressed significantly the expression of pentylenetetrazole-kindled seizures. Pretreatment with IGEFA caused dose-dependent increase in the latency of myoclonic jerks [F(7, 42) = 135.3, *p* < 0.001] as well as the latency to generalized tonic-clonic seizure [F(7, 38) = 54.1, *p* < 0.001] and a decrease in number of myoclonic jerks [F(7, 32) = 104.8, *p* < 0.001] as compared to the vehicle-treated pentylenetetrazole mice. IGEFA treatment produced a significant increase in the latency to myoclonic jerks from 45.4 ± 8.6 s in the vehicle-treated pentylenetetrazole mice to 126.5 ± 8.2 s (*P* < 0.001) and 173.6 ± 9.3 s (*P* < 0.001) in the groups administered IGEFA at the doses of 60 and 90 mg/kg, respectively. The results of protection offered by IGEFA 90 mg/kg were comparable to those of sodium valproate-treated group (Table 2). A significant difference in the number of myoclonic jerks was observed [F(7, 42) = 128.7, *p* < 0.001]. This decreased from 64.3 ± 2.5 in the vehicle-treated pentylenetetrazole mice to 18.5 ± 2.1 (*P* < 0.001), 14.2 ± 2.3 (*P* < 0.001) and 8.4 ± 1.2 (*P* < 0.001) in the groups administered IGEFA 30, 60 and 90 mg/kg, respectively (Table 2). Oral administration of IGEFA caused a significant difference in the onset of clonic seizures [F(7, 24) = 102.2, *P* < 0.001]. IGEFA significantly increased the onset of clonic seizures from 61.8 ± 8.6 s in the vehicle-treated pentylenetetrazole mice to 185.2 ± 9.2 s (*p* < 0.05), 285.2 ± 7.6 s (*p* < 0.01) and 371.5 ± 6.8 s (*p* < 0.001) in IGEFA 30, 60 and 90 mg/kg treated groups, respectively (Table 2). IGEFA significantly increased the latency of generalized clonic-tonic seizure from 185.4 ± 15.6 s in vehicle-treated pentylenetetrazole mice to 324.2 ± 27.1 s (*P* < 0.05), 388.2 ± 35.3 s (*P* < 0.01), and 495.5 ± 57.4 s (*p* < 0.01) in the groups administered IGEFA 30, 60 and 90 mg/kg, respectively (Table 2). There was a significant difference in the duration of generalized tonic-clonic seizure amongst the different groups [F(7, 58) = 124.1, *p* < 0.001]. IGEFA decreased the duration of generalized tonic-clonic seizure from 19.5 ± 1.3 s in the vehicle-treated pentylenetetrazole mice to 8.2 ± 0.2 s (*p* < 0.05), 4.9 ± 0.2 s (*p* < 0.01) and 3.3 ± 0.2 s (*p* < 0.001) in the groups administered 30, 60 and 90 mg/kg of the IGEFA, respectively (Table 2). Seizure stage amongst the groups was also significantly different [F(7, 43) = 195.3, *p* < 0.001]. The IGEFA significantly decreased the seizure mean stage from 4.0 ± 0.0 in the vehicle-treated pentylenetetrazole mice to 2.2 ± 0.3 (*p* < 0.05), 1.8 ± 0.3 (*p* < 0.01) and 1.3 ± 0.4 (*p* < 0.01), respectively in the groups administered IGEFA 30, 60 and 90 mg/kg, respectively (Table 2).

### Effects of IGEFA on brain MDA levels in pentylenetetrazole-kindled mice

The malondialdehyde level in the vehicle-treated pentylenetetrazole mice was significantly higher than that seen in vehicle-treated saline mice. Pentylenetetrazole kindling significantly increased (*p* < 0.001) the brain malondialdehyde level from 142.8 ± 16.2 nmol/g wet tissue in the vehicle + saline-treated mice to 454.6 ± 18.4 nmol/g wet tissue in the distilled water-treated pentylenetetrazole mice. The IGEFA dose-dependently and significantly attenuated the increased brain malondialdehyde levels due to pentylenetetrazole kindling in a dose-dependent manner [F(7, 53) = 38.2, *P* < 0.001]. The MDA levels significantly decreased from 454.6 ± 18.4 nmol/g wet tissue in the vehicle-treated pentylenetetrazole mice to 195.1 ± 19.2 nmol/g (*p* < 0.05), 178.5 ± 17.4 nmol/g (*p* < 0.001) and 156.2 ± 16.4 nmol/g (*p* < 0.001) wet tissue in groups administered IGEFA 30, 60 and 90 mg/kg, respectively. IGEFA per se (90 mg/kg) caused a decrease in the oxidative stress as indicated by the significant decrease (*p* < 0.001) in whole brain malondialdehyde levels as compared with the vehicle-treated pentylenetetrazole mice (Table 3).

**Table 3:** Effects of IGEFA on lipid peroxidation product, brain reduced glutathione and brain GABA content in whole mice brain on pentylentetrazole-induced kindling in mice

Treatments	Dose (mg/kg)	Brain lipid peroxidation, MDA (nmol/g-wet tissue)	Brain reduced glutathione, GSH (μg/g-wet tissue)	Brain GABA level (μg/g-wet tissue)
Vehicle + Saline	– + –	142.79 ± 16.24	196.24 ± 12.31	398.51 ± 12.42
Vehicle + PTZ	– + 30	454.62 ± 18.41	101.17 ± 14.24	297.38 ± 13.59
IGEFA + PTZ	15 + 30	362.24 ± 16.26*	132.15 ± 13.15*	398.53 ± 18.37
IGEFA + PTZ	30 + 30	195.13 ± 19.15**	158.47 ± 12.41*	397.62 ± 13.85
IGEFA + PTZ	60 + 30	178.47 ± 17.39**	185.75 ± 14.53**	435.49 ± 17.52*
IGEFA + PTZ	90 + 30	156.19 ± 16.43***	192.25 ± 16.24**	455.13 ± 16.38**
SVA + PTZ	300 + 30	149.17 ± 19.75***	195.62 ± 14.46**	452.36 ± 15.46**
SVA + Saline	300 + –	147.36 ± 17.15***	197.37 ± 19.31**	462.37 ± 17.25**
IGEFA + Saline	90 + –	152.61 ± 17.35***	199.21 ± 14.19**	471.41 ± 14.33**

Results are expressed as mean ± S.E.M., for 6 animals. Data were analysis by two-way ANOVA, followed by Newman-Keuls post hoc test

*MDA* malondialdehyde, *GSH* glutathione, *GABA* gamma-aminobutyric acid, *SVA* sodium valproate, *IGEFA* iridoid glycosides extracts of *Feretia apodenthera*

*P < 0.05, **P < 0.01, ***P < 0.001, significantly different compared to the vehicle-treated PTZ mice

### Effects of IGEFA on brain glutathione levels in pentylenetetrazole-kindled mice

The marker for oxidative stress, glutathione, plays an important role in protecting cells against oxidative damage by scavenging free radicals. Thus, in the present study, whole brain glutathione levels were measured in all mice groups. There were significant differences in reduced glutathione levels between the test groups and the negative control group [F(7, 54) = 102.3, *P* < 0.001]. In vehicle-treated pentylenetetrazole animals the brain glutathione level was 101.2 ± 14.3 μg/g-wet tissue. The IGEFA (15, 30, 60 and 90 mg/kg, p.o.) administration in all doses significantly increase this levels. The glutathione levels are 185.8 ± 14.5 μg/g-wet tissue (*p* < 0.01) and 192.3 ± 16.2 μg/g-wet tissue (*p* < 0.01) for the mice pretreated by the doses of 60 and 90 mg/kg IGEFA, respectively. IGEFA (90 mg/kg) alone also caused a significant increase (199.2 ± 14.2 μg/g-wet tissue; *p* < 0.01) in the brain glutathione levels as compared to the control group (Table 3).

### Effects of IGEFA on brain GABA content

The systemic administration of the IGEFA (15–30 mg/kg, p.o.) did not produce any significant effect (*p* > 0.05) in the level of brain GABA concentration in animals (Table 3). However a significant increase in the level of brain GABA concentration in animals administered with the IGEFA was observed at the doses of 60 mg/kg (*p* < 0.01) and 90 mg/kg (*p* < 0.01), and sodium valproate (300 mg/kg, *p* < 0.001) the positive control, was observed 1 h after oral administration (Table 3).

### Acute toxicity

The general behavioral changes of the mice were observed following oral administration of IGEFA at 5, 15, 30, 90, 180 and 360 mg/kg doses, which were graded through time. The IGEFA at doses lower than 360 mg/kg in mice did not produce any abnormality in fur, eye color, asthenia, anorexia, salivation, piloerection, or diarrhea in all the treated mice, and there were no deaths recorded. Doses of 5–360 mg/kg did not cause any detectable changes, whereas, the doses of 720–5760 mg/kg seemed to be lethal and caused deaths within 24–48 h. The physical signs and symptoms of toxicity, which occurred in response to IGEFA, were a decrease in motor activity and exploration. The mice that died from a high dose (2880–5760 mg/kg) of IGEFA showed signs of respiratory failure (decreased respiratory rate and irregular breathing), gasping and coma before death. The internal organs of both controlled and treated groups did not show any unusual signs and were found to be normal in both size and color. The median lethal dose (LD_50_) was calculated to be 2197.7 mg/kg (Table 4).

**Table 4:** Acute toxicity of the IGEFA administered orally to different groups of mice

Treatment	Dose (mg/kg)	Sex	D/T	Mortality latency (h)	Toxic symptoms
Vehicle	–	Male	0/5	–	None
		Female	0/5	–	None
IGEFA	5	Male	0/5	–	None
		Female	0/5	–	None
IGEFA	15	Male	0/5	–	None
		Female	0/5	–	None
IGEFA	30	Male	0/5	–	None
		Female	0/5	–	None
IGEFA	90	Male	0/5	–	None
		Female	0/5	–	None
IGEFA	180	Male	0/5	–	None
		Female	0/5	–	None
IGEFA	360	Male	0/5	–	None
		Female	0/5	–	None
IGEFA	720	Male	3/5	36–48	Hypoactivity, piloerection, salivation
		Female	5/5	36–48	Hypoactivity, piloerection, salivation
IGEFA	1440	Male	4/5	24–36	Hypoactivity, piloerection, salivation
		Female	5/5	24–36	Hypoactivity, piloerection, salivation
IGEFA	2880	Male	5/5	24–36	Hypoactivity, piloerection, salivation
		Female	5/5	36–48	Hypoactivity, piloerection, salivation
IGEFA	5760	Male	5/5	36–48	Asthenia, anorexia, salivation, asthenia
		Female	5/5	36–48	Asthenia, anorexia, salivation, asthenia

D/T = Dead/Treated mice; None = No toxic symptoms during the observation period; mortality latency = time to death after the oral administration. The IGEFA was two groups of mice. Mice in each group were carefully examined for any signs of toxic (behavioural changes and mortality) for 14 days. Control group received vehicle (10 ml/kg, per os)

## Discussion

The present studies were undertaken in order to examine the anticonvulsant effects of IGEFA using mice models of generalised tonic-clonic seizures induced by chemicals. IGEFA has anticonvulsant effects on seizures triggered by the GABA_A_ receptor antagonists bicuculline or pentylenetetrazole. This anticonvulsant effects is demonstrated with both electroencephalographic recordings and behavioural observations.

Interestingly, the effects of IGEFA against bicuculline-induced motor seizures are comparable with the standard anticonvulsant diazepam in the case of acute administration. IGEFA (30–90 mg/kg) administered acutely by the oral route strongly protected mice against the convulsions induced by bicuculline, a selective antagonist of GABA at the GABA_A_-receptors [38]. Bicuculline, acts directly on the postsynaptic GABA_A_ receptor complex to induce hyperactivity behaviour and seizures [39]. GABA is the major inhibitory neurotransmitter of the mammalian nervous system and bicuculline, a competitive GABA_A_ receptor antagonist is a known pro-convulsant [40, 41]. The dose-dependent increase in the anticonvulsant effects of IGEFA in the bicuculline-induced motor seizures test may be explained by an action of IGEFA on the GABA_A_ receptor itself in the central nervous system. However, it may also act differently on other signalling contributors to counterbalance the excessive stimulation provided GABA_A_ antagonism.

It has been reported that pentylenetetrazole induces seizures by inhibiting the GABA pathway in the central nervous system [42–44]. In addition, altered excitatory, inhibitory and/or modulatory neurotransmitter receptor densities have been observed in the brains of pentylenetetrazole-treated animals [45]. IGEFA strongly protected mice against 70 mg/kg pentylenetetrazole-induced seizures. This antagonism of pentylenetetrazole-induced seizures suggested the existence of anticonvulsant activity and the interaction of the IGEFA with GABAergic neurotransmission or its functional neutralization [7, 46]. In addition, to determine which neurotransmitter system is involved in the acute anticonvulsant properties of IGEFA, a selective benzodiazepine receptor antagonist, flumazenil or FG7142, an inverse diazepam receptor agonist of the GABA_A_ receptor complex, were introduced in the 70 mg/kg pentylenetetrazole-induced seizures test. Mice protection by the IGEFA administration at a dose of 90 mg/kg was indeed antagonized by flumazenil or FG7142. These results indicate that the effects of IGEFA should be mainly mediated via the GABAergic system [26, 38, 47].

Pentylenetetrazole administration produces a characteristic behavioral pattern of events: ear twitch, vibrissae twitch, straub tail, myoclonic twitch, forelimb clonus, falling and tonic hind limb extension [22]. These seizure behaviors correlate with spiking activity and spike-wave discharges in the cortex as measured by electroencephalography (EEG) [22]. Pre-treatment with IGEFA significantly attenuated the 50 mg/kg pentylenetetrazole-induced seizures in mice. The pentylenetetrazole-induced epilepsy model is an effective model for screening the efficacy of anti-epileptic drugs. The behavioral and electroencephalographic manifestations of pentylenetetrazole-induced seizures in rodents suggest that the studied test is a model of generalized tonic-clonic seizures [21]. The acute administration of IGEFA at the doses of 30, 60 and 90 mg/kg significantly suppressed hippocampal and cortical paroxysmal discharges in a dose-dependent way; and the number of crisis, the cumulative duration of crisis, and the mean duration of crisis recorded were significantly decreased or partially blocked. In the current study it was found that the time elapsed before the appearance of myoclonic jerk and generalized tonic-clonic seizures was significantly increased in mice treated with various doses of IGEFA. Our results demonstrate that inhibition of excitatory drive in the cortex and hippocampus can significantly delay seizures onset and suppress epileptiform discharges. This provides a proof of principle that this IGEFA could be used in the future for treatment of generalized tonic-clonic seizures and for management of generalized epilepsy. The anticonvulsant effects of IGEFA against pentylenetetrazole-induced seizures indicated their possible effectiveness also against absence seizures as drugs that inhibit pentylenetetrazole-induced convulsions are generally effective against absence seizures [48, 49]. Pentylenetetrazole induces convulsions by inhibiting the GABA pathway in the central nervous system via the inhibition of GABA_A_ receptor-chloride channel complex [7, 49–51]. However, the effects of a lyophilized aqueous extracts stem barks extracts of *Feretia apodanthera* against pentylenetetrazole-induced kindling, -induced kindled seizures, or prolongation of hexobarbital sleeping time have been reported [10, 18].

In the present study, sub-convulsive dose of pentylenetetrazole, when given intraperitoneally, on alternate days induced kindling in the vehicle-treated mice. The groups which were administered IGEFA showed dose-dependent protection against seizures. Kindled seizures are widely accepted as an animal model of epilepsy, wherein repeated sub-threshold brain stimulation, electrical or chemical, leads to behavioral signs of tonic and clonic seizures [52]. IGEFA significantly increased the latencies to myoclonic jerks, clonic seizures and generalized tonic-clonic seizure as well as duration of generalized tonic-clonic seizure as compared to the vehicle-treated pentylenetetrazole mice. The number of myoclonic jerks was also decreased by IGEFA in a dose-dependent manner. IGEFA also decreased the mean seizure stage dose-dependently as compared to the vehicle-treated pentylenetetrazole mice. IGEFA produced maximum seizure protective effect in the dose of 90 mg/kg. The seizure protection offered by IGEFA (90 mg/kg, p.o.) was comparable to the standard antiepileptic drug sodium valproate (300 mg/kg, p.o.). The protection offered by valproate acid on pentylenetetrazole-induced kindling is well established [53]. Sodium valproate, as a standard anticonvulsant drug, has anticonvulsant effects on seizure induced by pentylenetetrazole. Previous studies showed that sodium valproate is able to increase brain GABA levels via various mechanisms, including blocking GABA reuptake, inhibiting the enzymes that break down GABA, and enhanced GABA release from nerve terminals (central nervous system synapses) [54, 55]. The protective effect of sodium valproate on pentylenetetrazole-induced kindling is believed to be achieved through different neural mechanisms including inhibition of the voltage-dependent sodium channels, facilitation of GABAergic neurotransmission, reduced N-methyl-D-aspartate-receptor mediated glutamate excitation, increased serotonergic inhibition and attenuation of neurogenic inflammation [56–61].

Epilepsy has been described as a condition of excessive neuronal discharge associated with or resulting from oxidative stress [62, 63]. Free radicals are normal products of cellular aerobic metabolism involved in the development of seizures [64]. However, when the production of free radicals increases or defense mechanism of the body decreases, they cause cellular dysfunction by attacking at the polyunsaturated sites of the biological membranes causing lipid peroxidation. Lipid peroxidation was described as a main factor in the etiology of epilepsy. The increase in levels of malondialdehyde is a marker of lipid peroxidation [65]. In the present study, pentylenetetrazole kindling increased the level of malondialdehyde and decreased the glutathione level in the mice brain. Pentylenetetrazole thus caused an imbalance between antioxidant and oxidant defense system which may be at least partially responsible for seizures [66]. Oral administration of IGEFA prevented the rise in brain malondialdehyde levels in a dose-dependent manner. The significant decrease in brain malondialdehyde levels with concomitant IGEFA administration as compared to pentylenetetrazole alone treated mice indicates an attenuation of lipid peroxidation. IGEFA administered at a dose of 90 mg/kg alone also significantly decreased the malondialdehyde level as compared to the vehicle-treated distilled water group which supports its antioxidant property. This antioxidant effect of IGEFA is coherent with its anticonvulsant effects if one assumes indeed that excess oxidation is a consequence of convulsions.

In addition, the present study demonstrated that IGEFA has protective effects on pentylenetetrazole-induced convulsions and also on oxidative damage associated with pentylenetetrazole. The decrease level of glutathione was observed in the distilled water-treated pentylenetetrazole mice. IGEFA administration in all doses demonstrated increase in glutathione level in kindled mice brain tissue. Glutathione is an endogenous antioxidant which gets converted to oxidized form. This oxidized form of reduced glutathione reacts with free radicals and prevent generation of most toxic hydroxyl radical [66]. Increased lipid peroxidation during kindling is independent of iron salts and excitotoxin. These results indicate that, during kindling, there is an excessive oxidative stress pertaining as a consequence of reduced glutathione levels [10, 67, 68]. These antioxidant effects of the extracts of *Feretia apodanthera* in pentylenetetrazole-kindled mice are supported by the findings of a recent study [10] wherein oral supplementation of a lyophilized aqueous extracts of *Feretia apodanthera* decreased the malondialdehyde, and increased the glutathione levels in brain mice. However, IGEFA treatment has restored the reduced glutathione level in the brain tissues of pentylenetetrazole-kindled mice.

The main action of the pentylenetetrazole-induced seizures is reducing GABA levels [44, 69]. Reports suggest that pentylenetetrazole-induced seizures presumably by impairing GABA-mediated inhibition by an action at the GABA_A_ receptors [70]. GABA is one of the important endogenous inhibitory neurotransmitters widely distributed in the central nervous system. Its reduction in the brain is associated with a number of neurological disorders (such as anxiety, depression and epilepsy) [70, 71]. The present study further demonstrates that, systemic administration of IGEFA produces significant changes in behaviour, such as sedation, hypoactivity and motor incoordination in mice which is indicative of either central nervous system depressant or muscle relaxant effects [36]. The post-synaptic GABA_A_ receptors are implicated in the inhibitory mechanism. Drugs that enhance GABA_A_ receptor-mediated inhibition, such as benzodiazepines, phenobarbital, valproate and felbamate, can be used to prevent the convulsions induced by pentylenetetrazole [48, 72]. It was found that administration of IGEFA and sodium valproate significantly enhanced the brain GABA concentration which again is suggestive of an anticonvulsant action of the extracts and the reference drug. Taken together, we suggest that the anticonvulsant action of IGEFA is correlated to an increase in GABA concentrations in the brain [30, 73]. This was also evidenced in the electroclinical study. Further studies may be warranted to elucidate which exact compound is responsible for these actions although it should be noticed that all five iridoid glycosides had very resembling chemical structures. It is therefore not impossible that they possess equivalent pharmacological properties.

We did not observe any mortality case including with the highest doses of IGEFA (range studied 5–360 mg/kg). Therefore, IGEFA may be considered to be relatively safe at these doses.

## Conclusions

In summary, this study illustrated that IGEFA possess potent anticonvulsant activity and significantly reduces the progression of kindling and attenuates the oxidative stress in mice. These finding provides scientific rationale for the use of *Feretia apodanthera* extracts for the amelioration of generalized epilepsy observed in traditional medicine in some countries of Western Africa, particularly in Cameroon. Thus, more studies are necessary to clarify the chemical structural requirements involved in the antiepileptic effects of the IGEFA components and the biophysical mechanisms underlying their pharmacological properties directly on expressed GABA_A_ receptors.

## Abbreviations

ANOVA, analysis of variance; GABA, gamma-aminobutyric acid; HMQC, heteronuclear multiple quantum coherence; HN,: Herbier National du Cameroun; IGEFA, iridorid glycosides of *Feretia apodanthera*; NMR, nuclear magnetic resonance; RP-HPLC, reversed-phase high performance liquid chromatography
